# Overdose-Related Trends in Online Search Behavior in Japan: Analysis Using Infodemiological Methods

**DOI:** 10.2196/73794

**Published:** 2025-08-06

**Authors:** Miyu Eguchi, Soichiro Ushio, Satoru Esumi, Yukiomi Eguchi, Toshinobu Hayashi, Taisuke Kitamura, Kenichi Mishima, Takashi Egawa

**Affiliations:** 1 Department of Emergency and Disaster Medical Pharmacy, Faculty of Pharmaceutical Sciences, Fukuoka University, 8-19-1, Nanakuma, Jonan-ku, Fukuoka, 814-0180, Japan, 81 928716631; 2 Department of Clinical Drug Evaluation, Faculty of Pharmaceutical Sciences, Kobe Gakuin University, Kobe, Japan; 3 Department of Physiology and Pharmacology, Faculty of Pharmaceutical Sciences, Fukuoka University, Fukuoka, Japan; 4 Department of Emergency and Critical Care Medicine, Faculty of Medicine, Fukuoka University, Fukuoka, Japan

**Keywords:** search engine, infodemiology, over-the-counter, overdose, drug-related search behaviors, overdose–related online search trends

## Introduction

Overdose incidents involving over-the-counter (OTC) and prescription drugs have increased worldwide with the COVID-19 pandemic [1]. In Japan, overmedication of OTC drugs has become a major issue. With digital platforms' essential role in health information access, increase in drug overdose information has raised concerns [2,3]. Although infodemiological research has examined drug-related search behaviors [3], it does not capture the postpandemic increase in overdoses. This study aimed to investigate overdose-related online search trends in Japan using infodemiological methods.

## Methods

### Data Sources

Online search data on Yahoo! JAPAN, one of the most frequently used search engines in Japan [4], were obtained from DS.INSIGHT (last accessed: January 6, 2025). This platform provides data on search behaviors over time among different demographic groups, including search volume by sex. The search volume obtained from DS.INSIGHT is based on the number of users searching Yahoo! JAPAN and is extrapolated using the Ministry of Internal Affairs and Communications’ Telecommunications Usage Trends Survey. Online search data for “O-ba-do-zu” (overdose) from 2020 to 2024 were extracted. To identify drugs of interest, related queries were extracted; only terms with volumes exceeding 100 were included. Searches within one week before and after the overdose-related queries were analyzed. The identified drugs were categorized accordingly. Additionally, Japan’s weekly search data for the same timeframe were extracted using Python (version 3.11.3) with the Google Trends (GT) application programming interface (last accessed: May 30, 2025). “O-ba-do-zu” was queried as a search term, not a topic; the category was set to “all categories.” GT provides the relative search volume (RSV) on a 0‐100 scale, reflecting public interest in specific terms.

## Results

**Figure 1.**
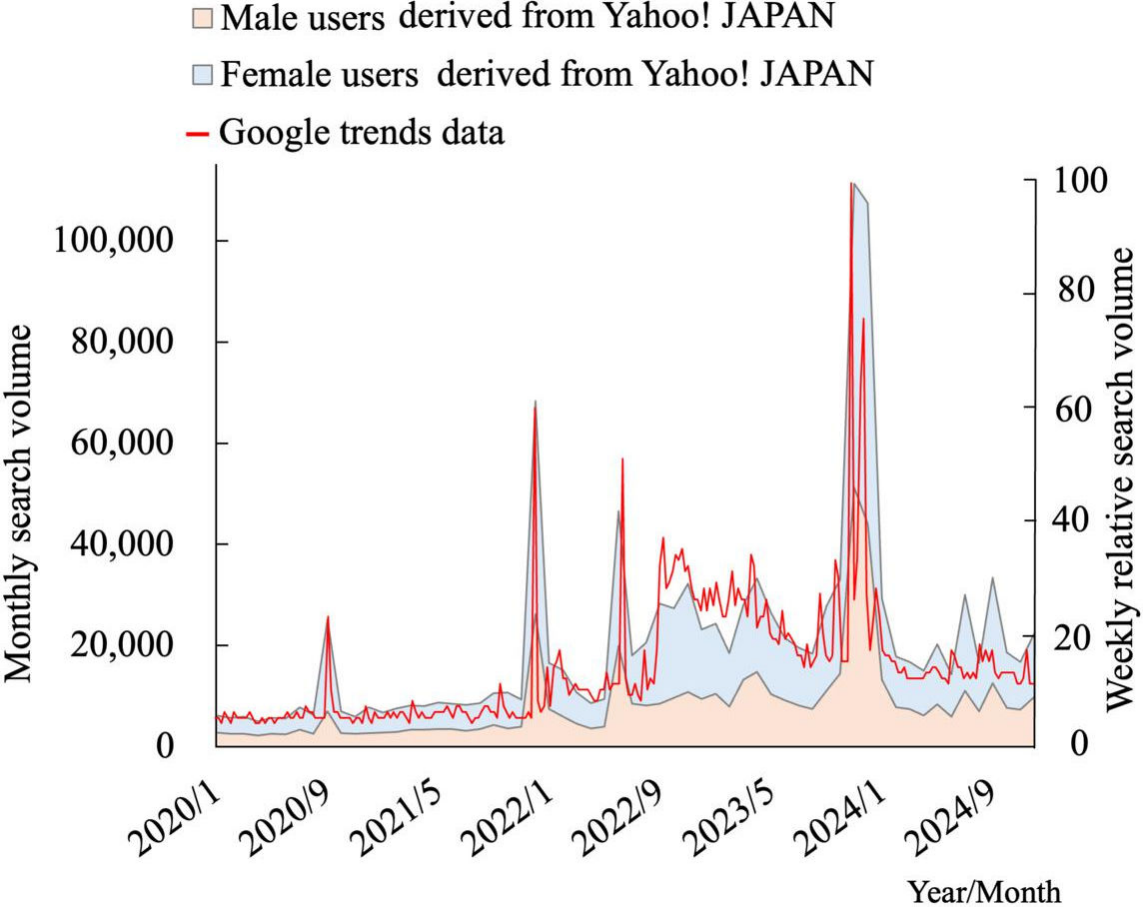
Search trends for “O-ba-do-zu (overdose)” in Japan, 2020‐2024.

The monthly search volume for “O-ba-do-zu” from DS.INSIGHT and weekly RSV for “O-ba-do-zu” from GT for 2020‐2024 are shown in Figure 1. DS.INSIGHT data showed that “O-ba-do-zu” queries in 2024 (240,000) were approximately five times greater than in 2020 (89,800). GT data showed that RSV for “O-ba-do-zu” increased during 2020‐2024. Searches by female users accounted for 52.67% (14,800/28,200)‐72.11% (18,100/25,000) of the search volume. Notable peaks in search frequency occurred in September 2020, December 2021, June 2022, and November-December 2023. Table 1 shows the frequency of drugs searched together with “O-ba-do-zu” in 2024, according to Yahoo! JAPAN. Among duplicate overdose-related searches, 11.8% (3100/26,300) were for OTC drug compounds, which included components of respiratory medications, sedative-hypnotics, and other categories.

**Table 1.** Drug classes queried by searchers for information on overdose. Classification of drugs searched within 1 week before and after “O-ba-do-zu” searches on Yahoo! Japan in Japan in 2024.

Drug class	Duplicate search volume (N=25,490), n (%)
Benzodiazepines and non-benzodiazepines	9320 (36.6)
Antipsychotics	9010 (35.3)
Antidepressants	2800 (11.0)
Respiratory drugs	2220 (8.7)
Mood stabilizers (eg, lithium and valproic acid)	1000 (3.9)
Other sedative-hypnotics (eg, bromvalerylurea and allylisopropylacetylurea)	580 (2.3)
Illegal drugs	560 (2.2)
Others (eg, potassium cyanide and herbal medicines)	810 (3.2)

## Discussion

In this study, we investigated overdose–related online search trends in Japan by using data from two search engines using infodemiological methods, and found increased online interest in drug overdoses in Japan over the past 5 years. We also elucidated the patterns of overdose–related search behavior and the specific interests of individuals conducting such searches. Our findings, including the higher proportion of female searchers [5,6] and ranking of searched drugs [7], are consistent with previous ones.

Seasonality in intentional drug overdose has been reported [8]. However, differing seasonal patterns were reported in another study [9], and a consensus remains to be established. Although we observed two sharp peaks in December over the 5-year period, no consistent periodic pattern was identified. In Japan, search spikes often coincided with socially and culturally impactful events, such as the release of a song titled “Overdose” in September 2022 and reports of overdose cases involving elementary school students.

In drug intoxication cases, multiple drugs are often consumed. While our results were consistent with previous ones, comparison of actual usage rates by using search volume data is difficult. Some compounds are used in both OTC and prescription drugs. Nevertheless, distinguishing OTC drugs from prescription ones by using search terms was difficult; therefore, we analyzed the retrieved compounds as OTC drugs. We found that OTC drug–associated compounds accounted for 11.8% of all retrieved drug-related searches, a proportion lower than 22.5% of OTC drug use, reported among transported patients. OTC drug overdoses are more common in younger individuals. As younger individuals tend to gather information from social media rather than Internet searches, these results may reflect a demographic bias toward older age groups [10]. This study analyzed search behavior, not actual overdose cases. Thus, the findings reflect public interest, rather than incident frequency. The main limitation of this study is the inability to demonstrate a direct link between search volume and real-world overdose events. Therefore, our findings should be interpreted cautiously.

In conclusion, rising interest in overdosing in Japan reflects growing public concerns. Monitoring trends and offering timely information may support early public health interventions and overdose prevention.

## Abbreviations

GT: Google Trends
OTC: Over-the-counter
RSV: relative search volume
